# Proteasomal cysteine deubiquitinase inhibitor b-AP15 suppresses migration and induces apoptosis in diffuse large B cell lymphoma

**DOI:** 10.1186/s13046-019-1446-y

**Published:** 2019-11-06

**Authors:** Liling Jiang, Yuening Sun, Jinxiang Wang, Qingyan He, Xinmei Chen, Xiaoying Lan, Jinghong Chen, Q. Ping Dou, Xianping Shi, Jinbao Liu

**Affiliations:** 1 Guangzhou Municipal and Guangdong Provincial Key Laboratory of Protein Modification and Degradation State Key Laboratory of RespiratoryDisease, School of Basic Medical Science, Affiliated Cancer Hospital of Guangzhou Medical University, Guangzhou, Guangdong China; 2 The Molecular Therapeutics Program, Barbara Ann Karmanos Cancer Institute, and Departments of Oncology, Pharmacology and Pathology, School of Medicine, Wayne State University, Detroit, MI USA; 3 Sino-French Hoffmann institute, Guangzhou Medical University, Guangzhou, China

**Keywords:** B-AP15, Diffuse large B cell lymphoma, Apoptosis, Migration

## Abstract

**Background:**

The first line therapy for patients with diffuse large B cell (DLBCL) is R-CHOP. About half of DLBCL patients are either refractory to, or will relapse, after the treatment. Therefore, identifying novel drug targets and effective therapeutic agents is urgently needed for improving DLBCL patient survival. b-AP15, a selective small molecule inhibitor of proteasomal USP14 and UCHL5 deubiquitinases (DUBs), has shown selectivity and efficacy in several other types of cancer cells. This is the first study to report the effect of b-AP15 in DLBCL.

**Methods:**

Cell lines of two DLBCL subtypes, Germinal Center B Cell/ GCB (SU-DHL-4, OCI-LY-1, OCI-LY-19) and Activated B Cell/ABC (SU-DHL-2), were used in the current study. Cell viability was measured by MTS assay, proliferation by trypan blue exclusion staining assay, cellular apoptosis by Annexin V-FITC/PI staining and mitochondrial outer membrane permeability assays, the activities of 20S proteasome peptidases by cleavage of specific fluorogenic substrates, and cell migration was detected by transwell assay in these GCB- and ABC-DLBCL cell lines. Mouse xenograft models of SU-DHL-4 and SU-DHL-2 cells were used to determine in vivo effects of b-AP15 in DLBCL tumors.

**Results:**

b-AP15 inhibited proteasome DUB activities and activated cell death pathway, as evident by caspase activation and mitochondria apoptosis in GCB- and ABC- DLBCL cell lines. b-AP15 treatment suppressed migration of GCB- and ABC-DLBCL cells via inhibiting Wnt/β-catenin and TGFβ/Smad pathways. Additionally, b-AP15 significantly inhibited the growth of GCB- and ABC DLBCL in xenograft models.

**Conclusions:**

These results indicate that b-AP15 inhibits cell migration and induces apoptosis in GCB- and ABC-DLBCL cells, and suggest that inhibition of 19S proteasomal DUB should be a novel strategy for DLBCL treatment.

## Background

Diffuse large B cell lymphoma (DLBCL) is the most common non-Hodgkin’s lymphoma which is highly heterogeneous [1]. Gene expressional profiling classifies DLBCL into at least three distinct molecular subtypes: an activated B cell-like (ABC), a germinal center B cell-like (GCB), and a primary mediastinal B cell lymphoma (PMBCL) [2–4]. Most of DLBCLs belong to GCB and ABC subtypes, representing up to 41 and 35%, respectively [1]. GCB subtype is characterized by the activation of Bcl-2 and c-Myc [5, 6], while ABC subtype is featured by constitutively activation of NF-κB pathway [7]. Interestingly, in response to standard CHOP (Cytoxan, Hydroxyrubicin, Oncovin, and Prednisone) chemotherapy, GCB-DLBCL patients have a significantly better outcome with relatively favorable 5-year overall survival rates compared to ABC-DLBCL patients [8–10]. However, the molecular basis for these differential responses of these two DLBCL subtypes remains unknown. While researchers have been looking for subtype-specific therapies for ABC or GCB, until now, there is no success [11].

Our current research is related to the involvement of proteasome ubiquitin system in DLBCL development and therapy-resistance. 20S proteasome inhibitor bortezomib, which was approved as a single agent in patients with multiple myeloma (MM), was evaluated in clinical phase III studies in DLBCL [1, 12], but the toxicity and limitation of bortezomib have been observed [13]. Compared to traditional 20S proteasome inhibitors, targeting the particular deubiquitinase in the ubiquitin proteasome system is a more selective and less toxic therapy strategy.

Deubiquitinases (DUBs) are important regulators in protein degradation and have been suggested to play an important role in cancer development and therapy resistance [14, 15]. In mammalian cells, there are three DUBs present in the 19S proteasome: USP14, UCHL5 and Rnp11. USP14 and UCHL5 are not constitutive proteasome subunits but are reversibly associated with the Rpn1 and Rpn13 subunits of the 19S RP base, respectively, whereas Rnp11 is an important part of 19S proteasome structure and activity. Following the recruitment of poly-ubiquitin chain-tagged substrate protein locates to 19S, USP14 and UCHL5 trim ubiquitin chains from the distal end while Rnp11 performs cleaving entire chains from substrates, which would then obtain entry into the proteolytic chamber of 20S core region for substrate protein degradation [16, 17]. It has been reported that USP14 and UCHL5 are highly expressed in various tumors and play an important role in regulating oncogenic signaling [18–21]. A recent study, for instance, showed that USP14 and UCHL5 were detected in tumor cell cytoplasm in 77 and 74% of the DLBCL cases, respectively [22]. UCHL5 and USP14 should thus be considered as new targets in DLBCL therapy. It has been reported that b-AP15, a small molecule inhibitor of USP14 and UCHL5 [23], is able to induce apoptosis and overcome bortezomib resistance in multiple myeloma and Waldenstroms macroglobulinemia [24, 25]. The effect of b-AP15 on DLBCL, however, has not been evaluated.

In the current report, we investigated the anti-tumor activity of b-AP15 in DLBCL. We found that cells of both ABC- and GCB-subtypes were sensitive to b-AP15 treatment. Our results from both in vitro and in vivo studies suggested that b-AP15, by inhibiting the activities of USP14 and UCHL5 deubiquitinases, can suppress migration and induce apoptosis in GCB- and ABC-DLBCL cells. This study illustrates the potential of b-AP15 to be a candidate therapy for DLBCL, providing a basis for clinical evaluation.

## Materials and methods

### Chemicals and reagents

b-AP15 was purchased from Merk Millipore (Darmstadt, Germany). The proteasome inhibitor, bortezomib (PS341), was purchased from BD Biosciences (San Jose, CA).SKL2001, IWR-1-endo, TP0427735 HCl, and SIS3 HCl were from SelleckChemicals (Huston, TX). TGFβ1 was purchased from Peprotech. Suc-LLVY-AMC, Z-LLE-AMC, Boc-LRR-AMC were obtained from BostonBiochem (Cambridge, MA). These reagents were dissolved in dimethyl sulfoxide (DMSO) as a stock solution, and stored at − 20 °C. In all experiments, final concentration of DMSO did not exceed 0.3%. Antibodies to the following proteins were purchased from Cell Signaling Technology (Danvers, MA) and used at a dilution of 1:1000: poly adenosine diphosphate ribose polymerase (PARP) (clone 4C10–5, #9532), phospho-Erk1/2 (T202/Y204, #4370), Erk1/2 (#4348), phospho-Akt (#2965), Akt (#4685), p27 (#3688), XIAP (#2045), caspase-8 (#9746), caspase-9 (#9504), Cleaved Caspase-3(9661S), apoptosis-inducing factor (AIF) (#5318), Bax (#5023), phospho-STAT5A/B (Y694/Y699; clone 8–5-2, #9314) and STAT5 (#9358), Bcl-2 (15071S), Smad2/3 (8685S), p-Smad2/3 (8828S), Dvl2 (3224S), LRP6 (3395S), p-LRP6 (Ser1490; 2568S), β-Catenin (8480S), Snail (3879S), Slug (9585S), E-Cadherin (14472S), and N-Cadherin (14215S). Antibodies against ALK-5 (mab5871) was purchased from (Minneapolis, MN). Antibodies against ubiquitin (P4D1) (sc-8017), USP14 (SC-515812) and Ki-67 (sc-23,900) were from Santa Cruz Biotechnology (Dallas, Texas).. Antibodies against cleaved-caspase-3 (AV00021), cytochrome c (C5118) and survivin (S8191) were from Sigma-Aldrich (St. Louis, MO). Anti-UCH37/UCHL5 antibody (ab124931) was from abcam (Cambridge, MA). Anti-GAPDH (#60630) and anti-Actin (#0768) antibodies were from Bioworld Technology (Minnesota, USA). HRP-conjugated goat anti-rabbit (AP132P) and anti-mouse (12–349) antibodies were from Merk Millipore.

### Cell culture

The DLBCL cell lines SU-DHL-4, OCI-LY-1, OCI-LY-19 (GCB-DLBCL) and SU-DHL-2 (ABC-DLBCL) were purchased from ATCC (Manassas, VA) and incubated in RPMI 1640 medium (LifeTechnologies, Waltham, MA) supplemented with 10% fetal calf serum (Hyclone, Waltham, MA), 100 unit/ml penicillin, and 0.1 mg/ml streptomycin. Cells were incubated at 37 °C and in water vapor–saturated air with 5% CO_2_ at one atmospheric pressure.

### Cell viability assay

MTS assay (CellTiter 96 Aqueous One Solution reagent, Promega, Madison, WI) was used to measure cell viability. Briefly, 2 × 10^4^ cells in 100 μl were treated with b-AP15 for 48 h. Control cells received DMSO for a final concentration the same as the highest concentration of b-AP15 but less than 0.3% (v/v). Four hours before culture termination, 20 μl MTS was added to the wells. The absorbance density was read on a 96-well plate reader at wavelength 490 nm.

### Cell counting assay

SU-DHL-4 and SU-DHL-2 cells were seeded into 24-well plates (2 × 10^5^ cells/ml, 1 ml/well) and treated with various concentrations of b-AP15 for indicated duration. Then 0.4% trypan blue (Sigma-Aldrich) was added to count the number of live and dead cells under a light microscope.

### Cell death assay

DLBCL cells were treated with various concentrations of b-AP15 for 24 h. Apoptosis was determined by flow cytometry using Annexin V-fluoroisothiocyanate (FITC)/PI double staining (Sungene Biotech, TianJin, China). DLBCL cells were collected, washed with PBS and resuspended with binding buffer (Sungene Biotech). The cell preparation was then stained with Annexin V and PI following manufacturer’s protocol. Samples were analyzed using FACSCalibur flow cytometer and CellQuestPro software. The Annexin V/PI positive cells in the culture dish were also imaged with an inverted fluorescence microscope equipped with a digital camera (AxioObsever Z1, Zeiss, Germany).

### Western blot analysis

Whole cell lysates were prepared in RIPA buffer (1 × PBS, 1% NP-40, 0.5% sodium deoxycholate, 0.1% SDS) supplemented with 10 mM b-glycerophosphate, 1 mM sodium orthovanadate, 10 mM NaF, 1 mM phenylmethylsulfonyl fluoride (PMSF), and 1 × Roche Protease Inhibitor Cocktail (Roche, Indianapolis, IN). To detect the level of cytochrome C and AIF, the cytosolic fraction was prepared with a digitonin extraction buffer (10 mM PIPES, 0.015% digitonin, 300 mM sucrose, 100 mM NaCl, 3 mM MgCl2, 5 mM EDTA, and 1 mM PMSF). Western blotting was performed as we previously described [26], using specific primary antibodies as indicated and appropriate horseradish peroxidase (HRP)-conjugated secondary antibodies as indicated.

### Measurement of mitochondrial membrane permeability

The mitochondrial membrane potential of cells treated with b-AP15 or untreated were assayed by mitochondrial membrane potential kit (Sigma-Aldrich, St. Louis, MO), following manufacturer’s instruction. DLBCL cells were treated with various doses of b-AP15, and after 24 h the cells were harvested, prepared in 1 ml warm medium, and then 5 μl cationic hydrophobic mitochondrial potential dye was added. The cells were incubated for 30 min in a 5% CO2, 37 °C incubator. After centrifugation the cells were resuspended with 500 μl assay buffer, followed by monitoring the cells using a flow cytometry with ƛex = 635 nm, ƛem = 660 nm at APC channel.

### Proteasomal activity assay

The 20S proteasomal peptidase activities were measured using synthetic fluorogenic substrates. To evaluate in vitro proteasome inhibition, cells were lysed in ice-cold lysis buffer (25 mM Tris-HCl, pH 7.4) for 10 min. Equal amounts of protein from each sample were then treated with various concentration of b-AP15 for 30 min, and then incubated at 37 °C with specific fluorogenic substrates (25 μM) for 2 h in dark. The substrates used were Suc-LLVY-AMC for chymotrypsin-like activity, Z-LLE-AMC for caspase-like activity and Boc-LRR-AMC for trypsin-like activity. Fluorescence intensity was measured using a spectrophotometer at excitation of 350 nm and emission of 438 nm (Varioskan Flash 3001, Thermo,Waltham, MA).

### Real-time quantitative polymerase chain reaction (PCR)

Total RNA was extracted using TRIzol reagent (Invitrogen, Waltham, MA). After quantification by spectrophotometry, the first-strand cDNA was synthesized from 500 ng of total RNA with the RNA reverse PCR Kit (TaKaRa, Dalian, ShangDong). Then one-tenth of total cDNA was used for real-time PCR with the SYBR Premix Ex TaqIIKit (TaKaRa). The reaction used the ABI7500 Real-Time PCR System. Relative gene expression was analyzed by the Comparative Ct method with GAPDH RNA as endogenous control. The primers for real-time PCR are as follows:

- Bcl-2 forward, 5′-AACATCGCCCTGTGGATGAC-3′;
- Bcl-2 reverse, 5′-AGAGTCTTCAGAGACAGCCAGGAG-3′;
- c-Myc forward, 5′-GGAGGCTATTCTGCCCATTTG-3′;
- c-Myc reverse, 5′-CGAGGTCATAGTTCCTGTTGGTG-3′;
- P65 forward, 5′-ACCTCGACGCATTGCTGTG-3′;
- P65 reverse, 5′-CTGGCTGATCTGCCCAGAAG-3′.

### HA-Ub-VS assay

SU-DHL-4 and SU-DHL-2 cells were harvested after treatment with or without b-AP15 over 3 h. The cells lysed using DUB buffer (25 mM Tris-HCl, 20 mM NaCl, 5 mM MgCl_2_, 200 μM ATP), then added HA-Ub-VS (1 μM) and incubated in 37 °C 30 min. The samples were boiled with SDS-PAGE sample loading buffer and subjected to Western blot analysis.

### Cell migration assays

SU-DHL-4 and SU-DHL-2 cells were treated with indicated concentration of b-AP15, SKL2001, IWR-1-endo, TP0427735 HCl, SIS HCl, and TGFβ1for 24 h. Thereafter, 2 × 10^6^ cells/ml of two cell types were starved in serum-free RPMI 1640 medium for 1 h at 37 °C in 5% CO_2_. Cell suspensions (2 × 10^5^ in 100 μl) were added to the upper chambers with a pore size of 8 μm (Corning) and 600 μl of complete medium to the lower chambers. After the plate incubated 2–3 h at 37 °C in 5% CO_2_, the cells in the lower chamber were counted.

### Xenograft model

Nude Balb/c mice were bred at the animal facility of Guangzhou Medical University. The mice were housed in barrier facilities with 12 h light dark cycle, with food and water available ad libitum. Totally 3 × 10^7^ cells of SUDHL-4 and SU-DHL-2 cells were inoculated subcutaneously on the flanks of 5-week-old mice, each subtype include 12 mice. After inoculation for 5–6 days, 12 mice evenly separated to vehicle and b-AP15 group randomly, then treated with either vehicle (Cremophor EL: PEG400: saline = 2: 2: 4) or b-AP15 (5 mg/kg/day) for a total of 11 days. The tumor sizes were measured and tumor volumes were calculated by the following formula: a^2^ × b × 0.4, where “a” is the smallest diameter and “b” is the diameter perpendicular to “a”. Tumor xenografts were removed, weighed, stored and fixed at day 11 after treatment. All experiments were performed in conformity to relevant guidelines and regulations. All animal studies were conducted with the approval of the Institutional Animal Care and Use Committee of Guangzhou Medical University.

### Immunohistochemical staining (IHC)

Formalin-fixed xenografts were embedded in paraffin and sectioned using standard techniques. Tumor xenograft sections were immunostained for Ubs, Ki67 and p-Smad2/3.MaxVisionTM reagent (MaixinBiol, Fuzhou, FuJian) was applied to each slide following the manufacturer’s instructions. Color was developed with 0.05% diaminobenzidine and 0.03% H_2_O_2_ in 50 mmol/L Tris-HCl, pH 7.6, and the slides were counterstained with hematoxylin. A negative control for every antibody was also included for each xenograft specimen by substituting the primary antibody with preimmunize serum.

### Statistical analysis

All experiments were performed at least thrice, and the results were expressed as mean ± SD where applicable. GraphPad Prism 5.0 software (GraphPad Software) was used for statistical analysis. Comparison of multiple groups was made with one-way ANOVA followed by Tukey’s test or Newman-Kueuls test. Value of *p* < 0.05 was considered statistically significant.

## Results

### b-AP15 inhibits cell viability and proliferation in cell lines of GCB- and ABC-DLBCL

**Fig. 1:**
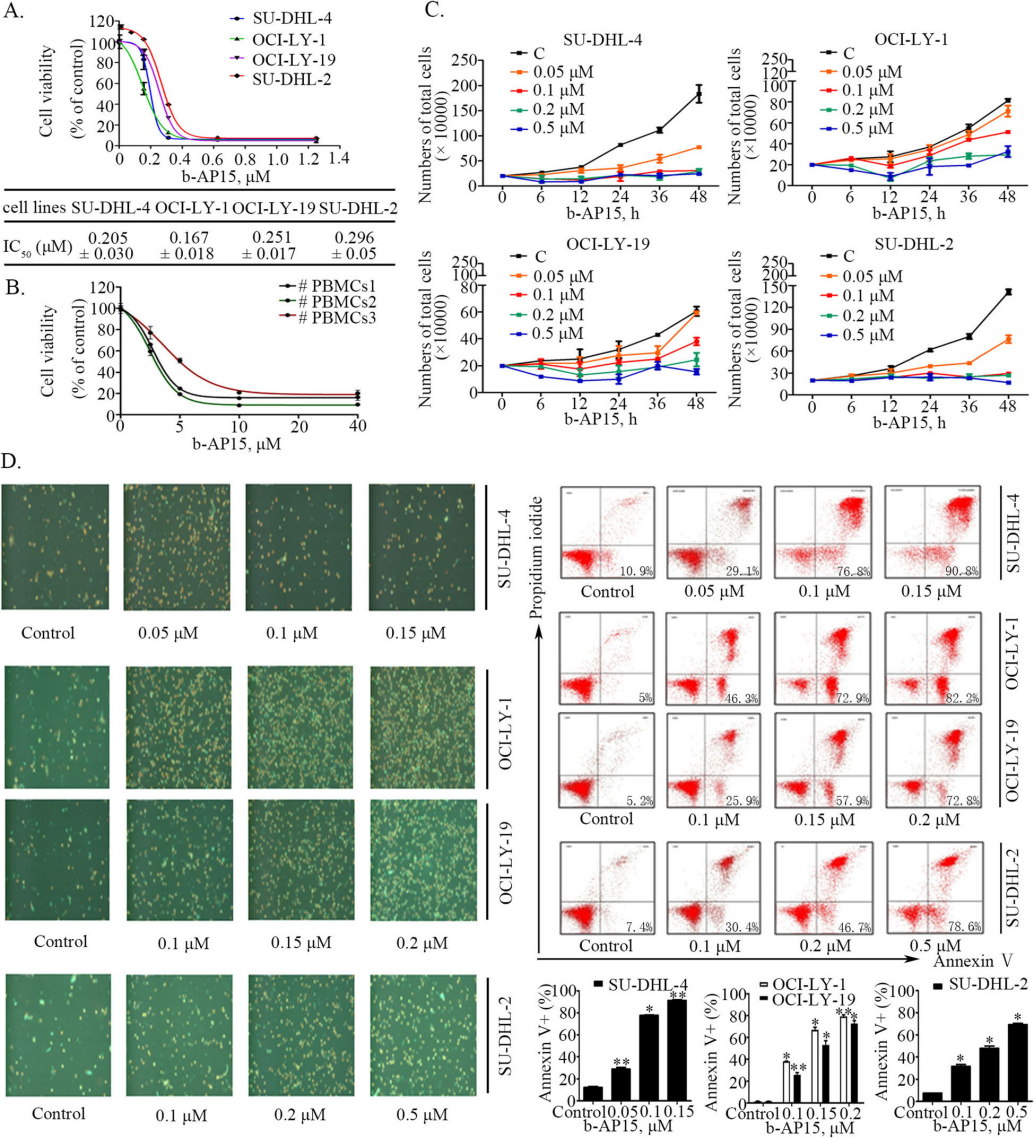
b-AP15 induces cell apoptosis and inhibits cell proliferation in two types of DLBCLs, ABC and GCB. (**a**) b-AP15 decreases cell viability of SU-DHL-2, SU-DHL-4, OCI-LY-1, OCI-LY-19 cells. DLBCL cells were cultured with b-AP15 at various concentrations for 48 h, and then were subjected to MTS assay. Mean ± SD(*n* = 3)(**b**) The cell viability of PBMCs from normal donors treated with b-AP15over 48 h. Mean ± SD(*n* = 3)(**c**) b-AP15 inhibits cell proliferation in both ABC and GCB-DLBCL cells. SU-DHL-2, SU-DHL-4, OCI-LY-1, OCI-LY-19 cells were planted in 24- well plates and treated with different doses of b-AP15 for 6 h, 12 h, 24 h. Then total cell number was detected by trypan blue exclusion staining. Mean ± SD (*n* = 3). (**d**) b-AP15 induces apoptosis in GCB- and ABC-DLBCL cells. SU-DHL-2, SU-DHL-4, OCI-LY-1, OCI-LY-19 cells were treated with showed concentrations for 24 h and apoptotic cells were detected by Annexin V-FITC / PI double staining, the images shown the Annexin V^+^/ PI^+^cells captured with inverted fluorescence microscope, and cell apoptosis was detected by flow cytometryexhibited by four-quadrant diagrams. The graphs were the statistics of the flow cytometry assay. Mean ± SD (*n* = 3). *P < 0.05, **P < 0.01, versus control group

To investigate the sensitivity of GCB- (SU-DHL-4, OCI-LY-1, OCI-LY-19) and ABC- (SU-DHL-2) DLBCL cells to proteasomal cysteine DUB inhibition, we treated the indicated cell lines with b-AP15 at various concentrations for 48 h, followed by measuring cell viability by MTS assay. As shown in Fig. 1a, b-AP15 dose-dependently decreased cell viability of all the lines of GCB- and ABC-DLBCL, with IC_50_ values of 0.205, 0.167, 0.251 and 0.296 μmol/L for SU-DHL-4, OCI-LY-1, OCI-LY-19 and SU-DHL-2, respectively. These results indicate that both ABC- and GCB-DLBCL cells have similar sensitivity to b-AP15. It should be noted that normal cells, PBMCs from health donors are much less sensitive to b-AP15 (IC_50_ ranging from 5.8 μmol/L to 10.4 μmol/L) than DLBCL cells (Fig. 1b). These data suggest that b-AP15 is much more selective to DLBCL cells than to normal cells.

We then performed a trypan blue exclusion assay to confirm the capacity of b-AP15 to inhibit proliferation in the two subtypes of DLBCL cell lines. As shown in Fig. 1c, b-AP15 decreased cell growth in a dose- and time-dependent manner.

### b-AP15 induces cell death in both GCB- and ABC-DLBCL cell lines

We next assessed the cell death-inducing ability of b-AP15 in GCB- and ABC-DLBCL cells by using Annexin V/PI staining assay. After SU-DHL-4, OCI-LY-1, OCI-LY-19 and SU-DHL-2 cell lines were treated with different concentrations of b-AP15 for 24 h, a significant increase of Annexin V^+^/PI^+^ cell populations was detected by inverted fluorescence microscopy, as shown in left panel of Fig. 1d. Similar results were obtained by flow cytometry analysis (Fig. 1d, right panel), confirming that b-AP15 triggered cell death in the two subtypes of DLBCL in a dose dependent manner.

### b-AP15-induced apoptosis was associated with activation of caspase and inhibition of anti-apoptotic protein expression

**Fig. 2:**
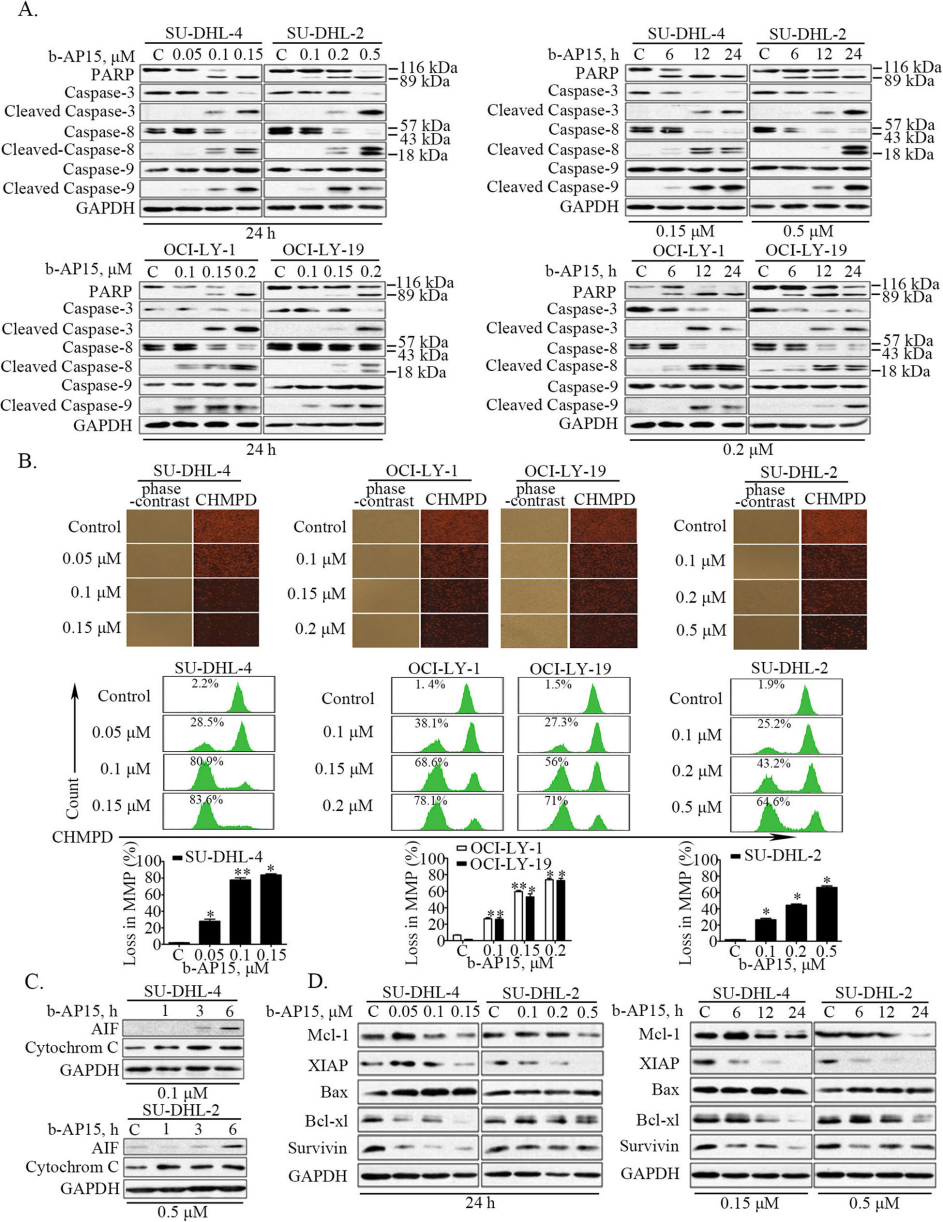
b-AP15-induced apoptosis was associated with activation of caspase and inhibition of anti-apoptotic protein expression in both GCB- and ABC-DLBCL cells. **a** b-AP15 induces cleavage of PARP and caspases-3, − 8, − 9 in SU-DHL-4, SU-DHL-2, OCI-LY-1, OCI-LY-19 cells. Cells were dose- and time-dependently treated with b-AP15, then PARP, and caspase-3, − 8, − 9 cleavage were analyzed by Western blots. GAPDH was used as a loading control. C, control. **b** b-AP-15 induces down regulation of mitochondrial membrane potential in SU-DHL-4, SU-DHL-2, OCI-LY-1, OCI-LY-19 cells. SU-DHL-4, SU-DHL-2, OCI-LY-1, OCI-LY-19 cells were treated with b-AP15 in different doses showed in data for 24 h, mitochondrial membrane potential were detected by inverted fluorescence microscope (the upper panel) or flow cytometry (the lower panel) after cationic hydrophobic mitochondrial potential dye staining. The fluorescence images exhibited the decrease of MMP which indicated with reduced red fluorescence. The results of flow cytometry assay were shown and the percentage of low MMP were labeled, and the statistics performed with graphs. Mean ± SD (*n* = 3). *P < 0.05, **P < 0.01, versus control group. **c** b-AP15 induces AIF and cytochrome C release. SU-DHL-4, SU-DHL-2 cells were exposed to b-AP15 for 3, 6, and 9 h, then cell cytoplasmic proteins were extracted and the released AIF and cytochrome C were detected by Western blot analysis. **d** b-AP15 decreases the expression of anti-apoptotic proteins in SU-DHL-4, SU-DHL-2 cells. Cells were dose- and time-dependently treated with b-AP15. The anti-apoptotic proteins Mcl-1, XIAP, Bax, Bcl-xl, Survivin were analyzed by Western blot analysis

To explore the mechanism of b-AP15-induced cell death, we measured the expression of several apoptosis-associated proteins. We found that b-AP15 markedly increased the cleavage of PARP, a hallmark of apoptosis (Fig. 2a). Consistently, b-AP15 activated caspase 3, caspase 8 and caspase 9 in a time- and dose-dependent manner (Fig. 2a). It has been documented that mitochondria plays a pivotal role in the regulation of cell apoptosis [27]. We next examined the effect of b-AP15 treatment on mitochondria. We found that the potential of mitochondrial membranes was decreased in DLBCL cell lines following the treatment with b-AP15, as shown by decreased levels of fluorescence intensity of CHMPD staining (top panel of Fig. 2b), and results of flow cytometry assay (bottom panel of Fig. 2b). Furthermore, the protein level of apoptosis inducing factor (AIF) and Cytochrome C in the cytoplasm was increased at earlier time points, suggesting that mitochondrial apoptosis pathway was activated in DLBCL cells after 19S cysteine DUB inhibition by b-AP15 (Fig. 2c). Moreover, we found that levels of several anti-apoptotic proteins, including Mcl-1, XIAP, Bcl-xl and Survivin, were significantly decreased after b-AP15 treatment in a dose- and time-dependent manner, while the protein level of pro-apoptotic Bax remain unchanged (Fig. 2d).

### b-AP15 inhibits proteasome function in GCB- and ABC-DLBCL cells

**Fig. 3:**
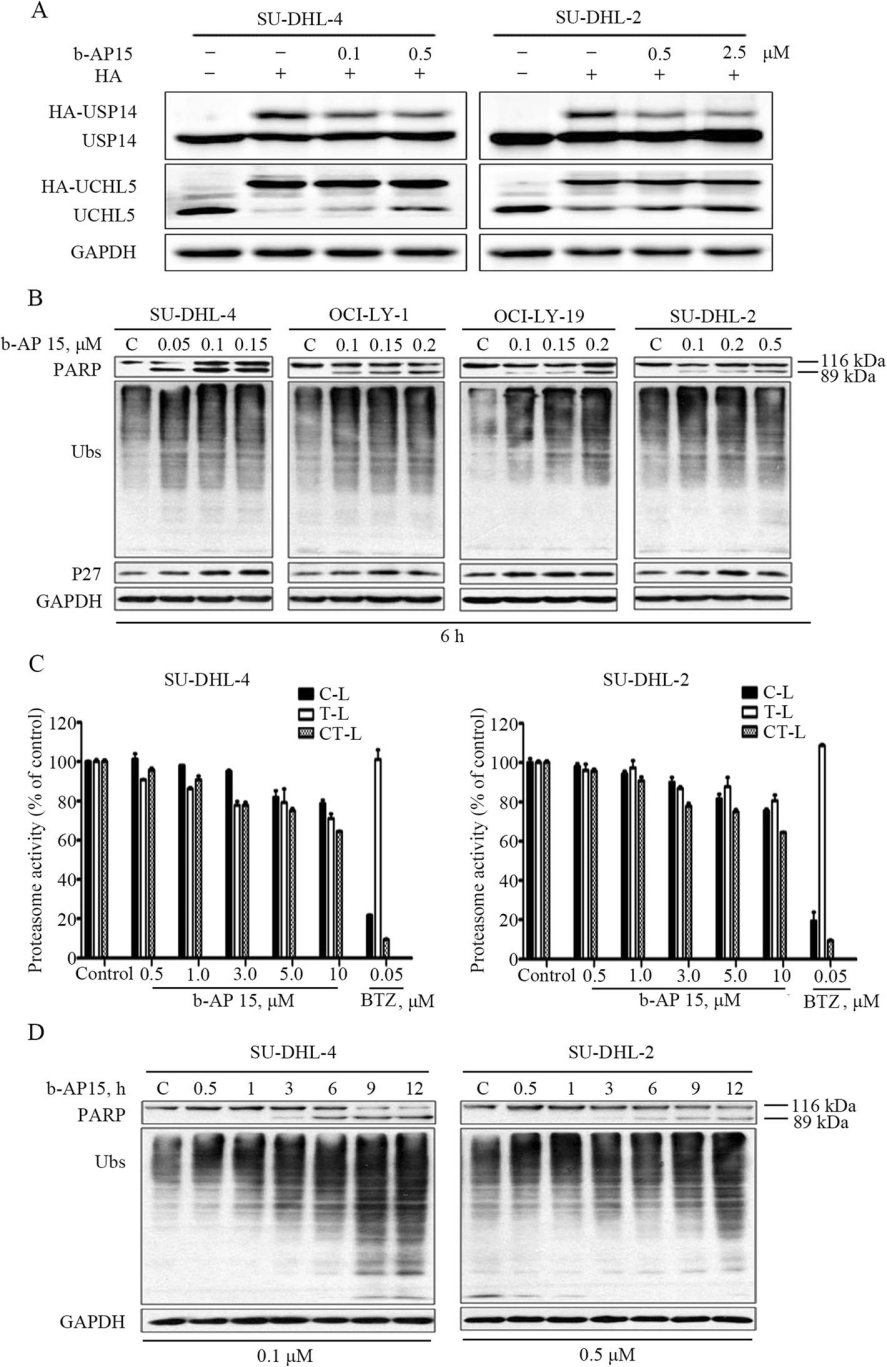
b-AP15 triggers cell apoptosis in ABC- and GCB-DLBCL by suppressing proteasome function. **a** b-AP15 inhibits USP14 and UCHL5 deubiquitinase activity in DLBCL cells. SU-DHL-4 and SU-DHL-2 cells incubated with b-AP15 over 3 h. The cells lysed with DUB buffer, then added 1 μM HA-Ub-VS and incubated in 37 °C 30 min. The protein levels of USP14 and UCHL5 were detected using western blot assay. **b** b-AP15 accumulates proteasome substrate proteins in DLBCL cells. Cells were treated with various doses of b-AP15 for 6 h. The protein levels of ubiquitin-proteins (Ubs) and p27 were detected using western blot assay. **c** b-AP15 has no obvious effect on the 20S proteasome peptidase activities in SU-DHL-4 and SU-DHL-2 cells. Cell lysate was treated with b-AP15, and then the C-like, T-like, CT-like activities at different times was recorded using the fluorogenic Z-LLE-AMC, Boc-LRR-AMC, Suc-LLVY-AMC substrates respectively. Mean ± SD (*n* = 3). **d** PARP cleavage occurs after proteasome inhibition with b-AP15 treatment. SU-DHL-4 and SU-DHL-2 cells were treated with the indicated dose of b-AP15 for the indicated duration. The protein levels of PARP and ubiquitin-proteins (Ubs) were detected with western blot assay

It has been reported that b-AP15 is a selective inhibitor of USP14 and UCHL5 [23]. However, the effect of b-AP15 in DLBCL has not been studied before. We performed the HA-Ub-VS assay in the selected DLBCL cell lines and confirmed that b-AP15 could competitively inhibit the interaction of HA-Ub-VS with USP14 or UCHL5 (Fig. 3a), which shows that b-AP15 impairs the deubiquitinase activity of USP14 and UCHL5. In addition, we found that b-AP15 dose-dependently induced accumulation of ubiquitin-proteins and proteasome substrate p27 protein in early time points (Fig. 3b). As the 26S proteasome is made of 19S regulatory complexes and 20S core portion, proteasome function could be injured when either portion has been targeted [23].. To confirm b-AP15 did not target the 20S proteasome peptidases in GCB- and ABC-DLBCL cells, we examined chymotrypsin-like, trypsin-like, and caspase-like proteasome activities, with 20S proteasome inhibitor bortezomib as a positive control. Our result confirms that b-AP15 did not impair activities of 20S proteasome in DLBCL cells (Fig. 3c). Accumulation of ubiquitinated proteins were observed as early as 1 h during the course of b-AP15 treatment (Fig. 3d). Importantly, obvious apoptosis-specific PARP cleavage was not observed until 6 h of b-AP15 treatment (Fig. 3b, d). These results indicate that the apoptosis induced by b-AP15 occurs after the proteasome inhibition.

### b-AP15 suppresses DLBCL cell migration

**Fig. 4:**
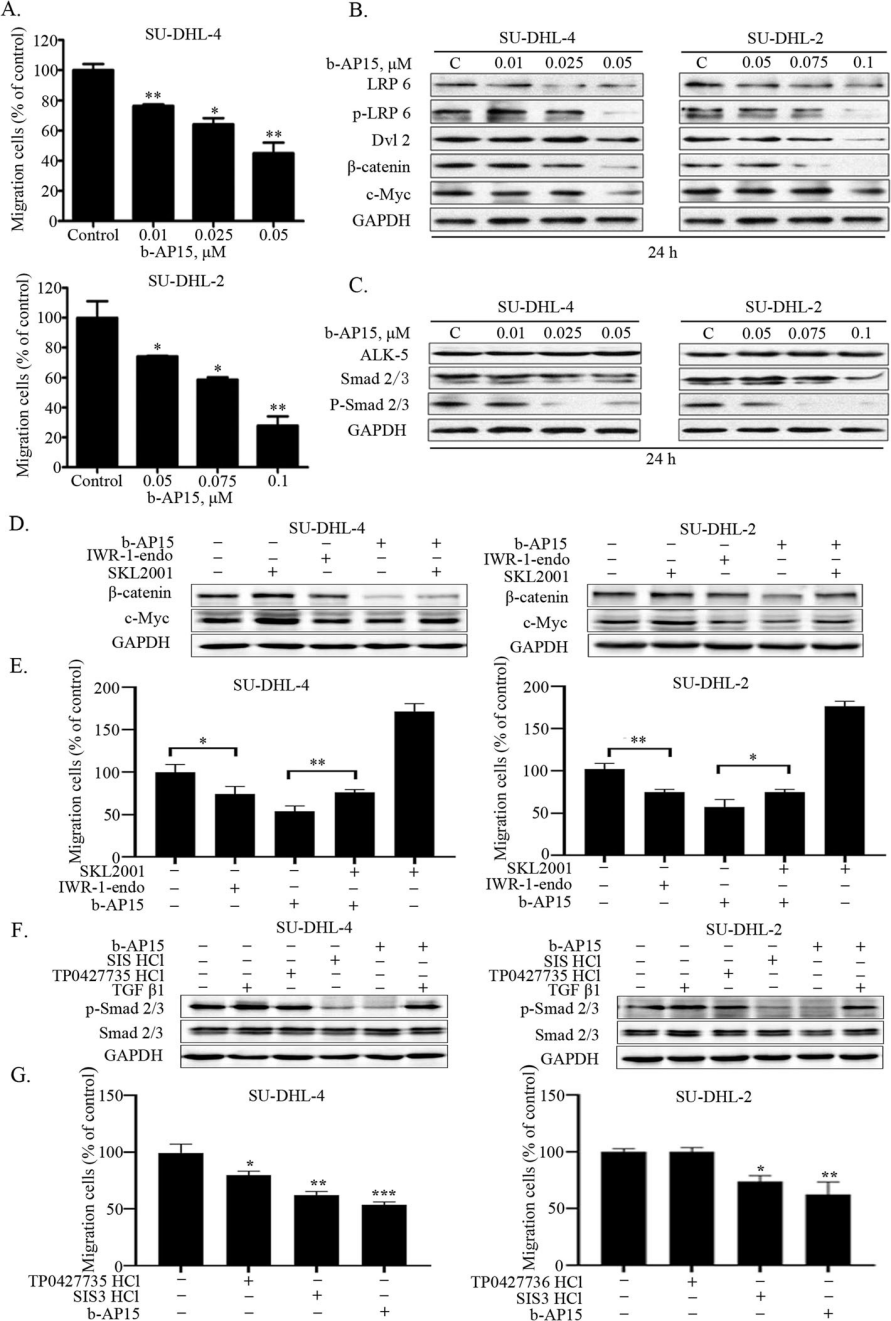
b-AP15 inhibits the migration of SU-DHL-4 and SU-DHL-2 cells through decreasingWNT and TGFβ canonical pathways. (**a**) SU-DHL-4 and SU-DHL-2 cells were dose dependently treated with b-AP15. The number of migration cells were decreased according to the raising doses. Mean ± SD (*n* = 3). * *P* < 0.05, **P < 0.01, ***P < 0.001 (**b**) WNT canonical pathway related proteins were analyzed by Western blot. Representative images were shown. (**c**) TGFβ canonical pathway related proteins were analyzed by Western blot. Representative images were shown. (**d**) The protein levels of β-catenin and c-Mycwere detected by Western blot with SKL2001(20 μM), IWR-1-endo(20 μM), or b-AP15(0.025 μM for SU-DHL-4 cellsand 0.075 μM for SU-DHL-2 cells) treatment over 24 h.Representative images were shown. (**e**) The cell migration assays were performed after SKL2001(20 μM), IWR-1-endo (20 μM) or b-AP15 incubated 24 h.Mean ± SD (*n* = 3). * *P* < 0.05, **P < 0.01, ***P < 0.001 (**f**) The protein level of p-Smad2/3 and Smad2/3 were detected by Western blot with 10 ng/ml rhTGFβ1, TP0427735 HCl (20 μM), SIS3 HCl (20 μM) or b-AP15 treatment over 24 h. Representative images were shown. (**g**) The cell migration was detected after treatment with TP0427735 HCl (20 μM), SIS HCl (20 μM) or b-AP15 over 24 h. Mean ± SD (n = 3). *P < 0.05, **P < 0.01, ***P < 0.0001, versus control group

Tumor metastasis is a significant cause of high mortality in DLBCLs. We then evaluated the effect of b-AP15 on migration in DLBCL cell lines. A dose-dependent decrease in number of migrated GCB- and ABC-DLBCL cells was observed when b-AP15 was used at low concentrations in the migration assay (Fig. 4a). According to the result, b-AP15 induced mild cell death even at the highest dose of migration in both cells (Additional file 1: Figure S1b). It is well known that Wnt/β-catenin and TGFβ/Smad are two essential pathways for tumor cell migration in through mediating the EMT signaling. Upregulation of CXC chemokine ligand 9 promotes viability and migration that can be abolished by knocking down of β-catenin in DLBCL cells [28], suggesting that Wnt/β-catenin signaling pathway plays an important role in DLBCL cell migration. Moreover, in Raji/ADM cells (B-cell non-Hodgkin’s lymphomas), inhibiting the activation of TGFβ signaling pathway via silencing Smad4 contributes to the suppression of cell viability, invasion and migration [29]. In addition, previous studies have shown that USP14 is an important regulator in Wnt/β-catenin signaling pathway by deubiquitinating Dvl [30]. Similarly, UCHL5 regulates deubiquitination of Smad2/3 and ALK-5 in TGFβ/Smad signaling pathway [31]. To verify the mechanism of b-AP15-mediated migration inhibition in DLBCL, we investigated the expression of these signaling molecules in SU-DHL-4 and SU-DHL-2 cells. The phosphorylation of LRP6, Dvl2, β-catenin and c-Myc was significantly decreased in a b-AP15-dose-dependent manner (Fig. 4b). We also found that the phosphorylation of Smad2/3 was also decreased in a dose-dependent manner (Fig. 4c). Furthermore, we activated TGFβ/Smad and Wnt/β-catenin signaling pathways by rh TGFβ1 and SKL2001, respectively. Two inhibitors TP0427735 HCl and SIS3 HCl were then used to suppress TGFβ/Smad signaling pathway, and IWR-1-endo was used to impair Wnt/β-catenin signaling pathway. Results of Fig. 4e show that the migration inhibition of b-AP15 could be mimicked by the β-catenin inhibitor IWR-1-endo and rescued by the agonist SKL2001 in DLBCL cells. Western blot assay in Fig. 4d demonstrated the corresponding changes of β-catenin and c-Myc protein levels with the same treatment. On the other hand, the smad3 inhibitor SIS3 HCl showed a significant suppression to cell migration, while TP0427736 HCl showed a relative weaker inhibition (Fig. 4g). Moreover, we observed that TGFβ induces p-Smad 2/3 and partially rescued the inhibition of p-Smad 2/3 by b-AP15 (Fig. 4f), while its effect on cell migration was not obvious (data not shown). Taken together, these data suggested that b-AP15 was able to inhibit the migration of GCB- and ABC-DLBCL cells via regulating Wnt/β-catenin and TGFβ/Smad signaling pathway.

### b-AP15 down-regulates molecular players involved in DLBCL progression

**Fig. 5:**
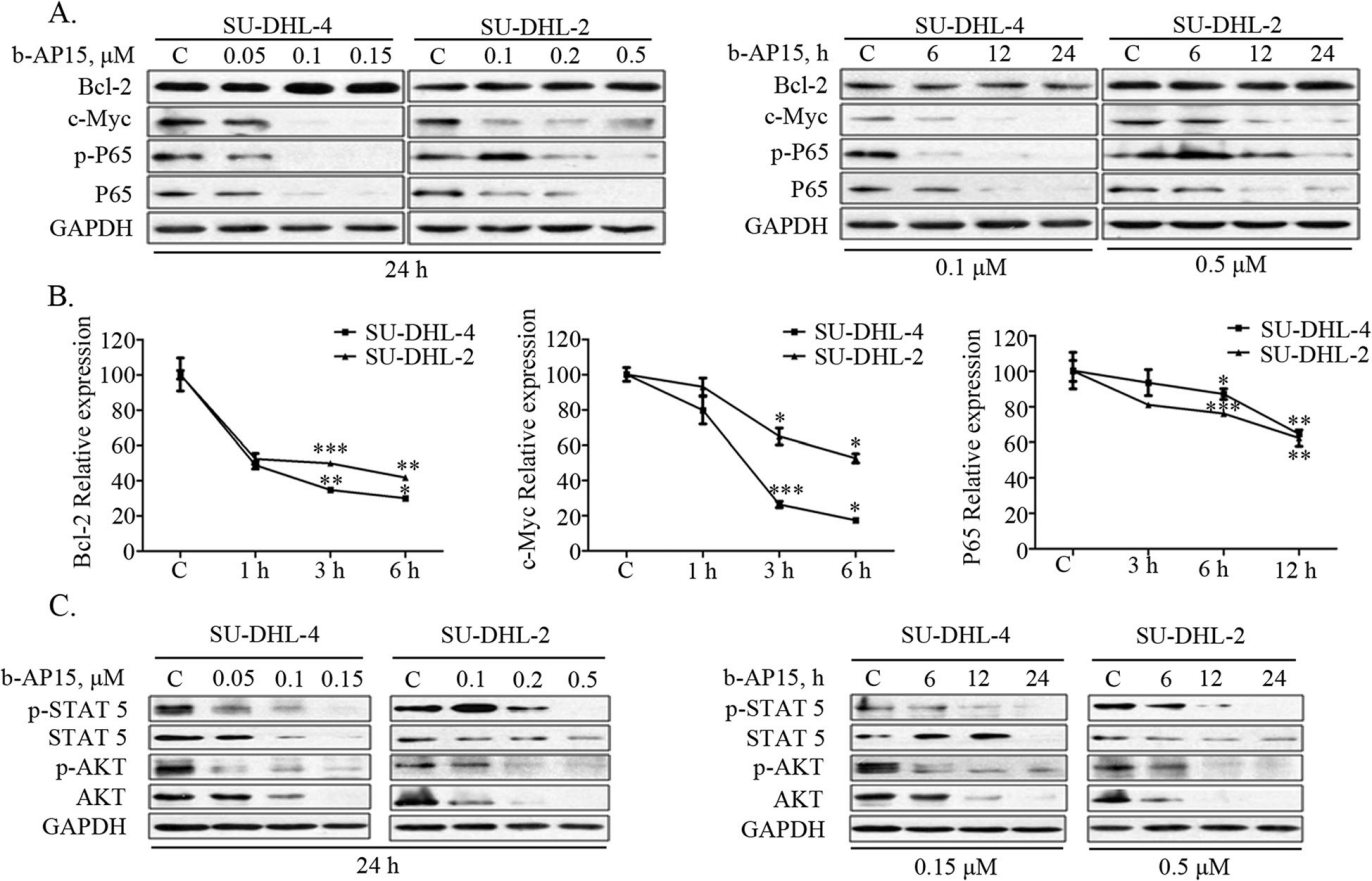
b-AP15 mediated down-regulation of molecules which were associated with progression in both GCB-and ABC-DLBCL cells. **a** b-AP15 decreases protein levels of c-Myc, NF-κB and phosphorylated NF-κB. Western blot analysis of DLBCL cells treated with b-AP15 as indicated both dose- and time-dependently was performed. **b** b-AP15 decreases mRNA expression of Bcl-2, c-Myc and NF-κB. SU-DHL-4 and SU-DHL-2 cells were treated for 3, 6 and 12 h with exposure to 0.1 μM and 0.5 μM b-AP15 respectively. The Bcl-2, c-Myc and NF-κB mRNA expression were measured by RT-qPCR and its expression level relative to the control was calculated. Mean ± SD (*n* = 3). *P < 0.05, **P < 0.01, ***P < 0.0001, versus control group. **c** b-AP15 decreases the protein levels of cell growth related signaling pathway AKT and STAT5. SU-DHL-4 and SU-DHL-2 cells weretreated with various concentrations or different duration of b-AP15. The total and phosphorylated AKT and STAT5 were analyzed by Western blot. Representative images were shown

GCB subtype of DLBCL is characterized with high protein levels of c-Myc and anti-apoptotic Bcl-2, whereas ABC-DLBCL subtype possesses the constitutive activation of NF-kB signaling [5–7]. We found that b-AP15 dose- and time-dependently inhibited the levels of total p65 protein, phosphorylated p65 and c-Myc but not Bcl-2 (Fig. 5a). Real-time PCR analysis detected significant decreases in the mRNA level of p65, c-Myc and Bcl-2 (Fig. 5b). We next evaluated the effects of b-AP15 on STATs and PI3K/Akt pathways which were associated with tumor cell growth and survival. The result showed that b-AP15 dose- and time-dependently inhibited phosphorylation of STAT5 and Akt (Fig. 5c), in agreement with our observation that b-AP15 has an inhibitory effect on cell proliferation of GCB- and ABC-DLBCL cells (Fig. 1c).

### b-AP15 restrains the growth of xenografted GCB- and ABC-DLBCL tumors in nude mice

**Fig. 6:**
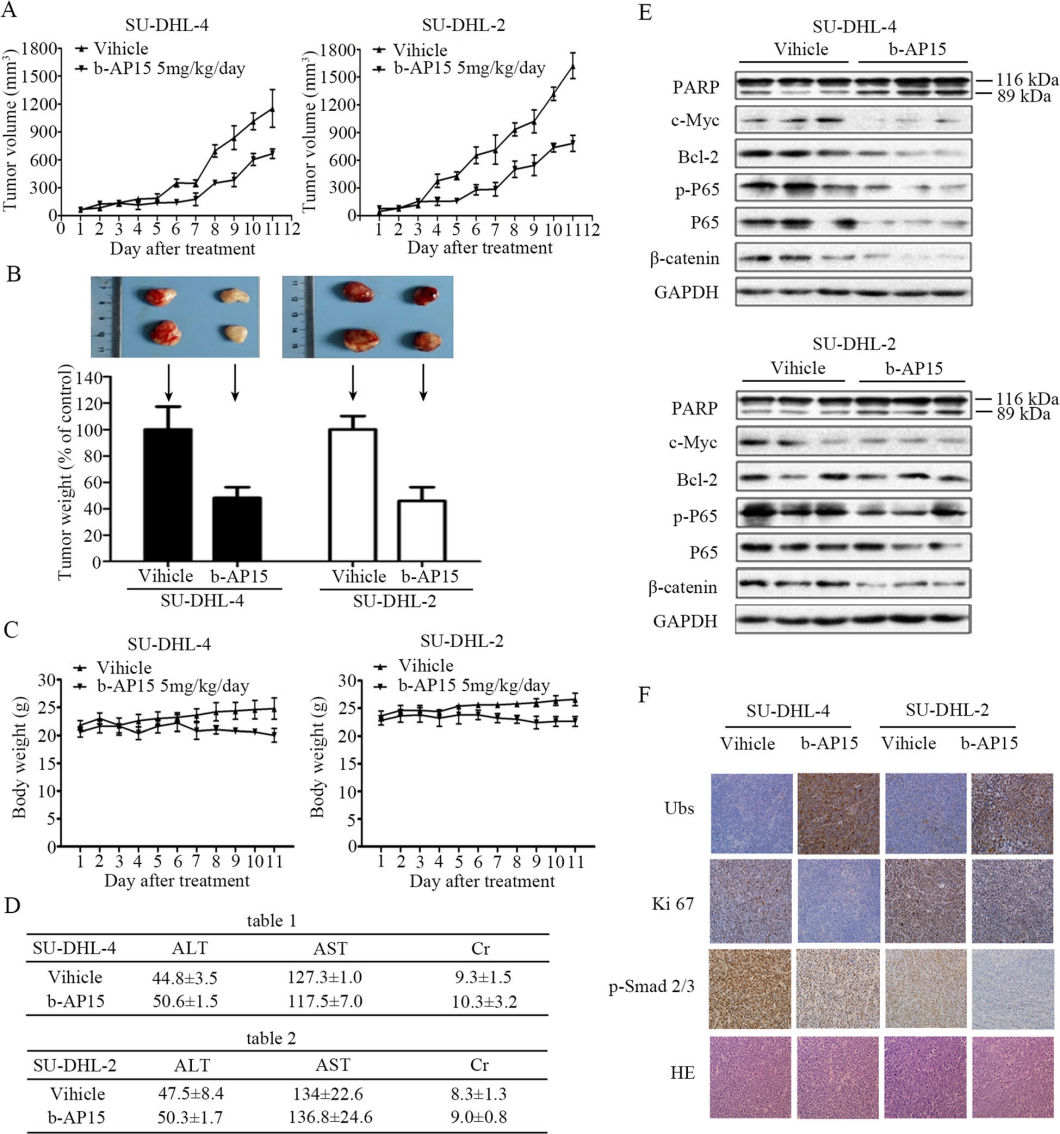
In vivo effect of b-AP15 in SU-DHL-4 and SU-DHL-2 cells derived mouse xenograft model. Nude mice bearing SU-DHL-4 and SU-DHL-2 cells were treated with either vehicle or b-AP15 (5 mg/kg/d) for 11 days after the average tumor size reached 50mm^3^. **a** b-AP15 inhibits tumor growth in vivo. Tumor growth curves were recorded every day in two sets of experiments. Mean ± SD (*n* = 6). *P < 0.05, **P < 0.01, ***P < 0.0001, versus b-AP15 treatment group. **b** On day 11 after inoculation, the mice were sacrificed, and the tumor tissues were weighed, imaged and summarized. *P < 0.05, **P < 0.01, ***P < 0.0001, versus control group. **c** Mice weights were recorded every day after b-AP15 treatment. Mean ± SD (*n* = 6). **d** The value of blood test indicators ALT (alanine aminotransferase), AST (aspartate aminotransferase), Cr (creatinine) were shown. Mean ± SD (*n* = 6). **e**, **f** The cell apoptosis and drug specific migration pathways related proteins in tumor tissues were detected by Western blot (SU-DHL-4 control group: #2, #4, #6; b-AP15-treated group: #13, #17, #18; SU-DHL-2 control group: #24, #28, #29; b-AP15-treated group: #32, #36, #37) and/or immunohistochemical (IHC) analysis. All the immunostaining and Western blot were repeated in three mouse tumor tissues and the most representative images were shown

To explore in vivo effects of b-AP15 on DLBCL tumors, we established nude mouse xenograft models by inoculating SU-DHL-4 and SU-DHL-2 cells subcutaneously. Mice with these DLBCL tumors were then treated with vehicle or b-AP15 (5 mg/kg/day, intraperitoneal injection) for 11 days. We found that b-AP15 treatment significantly inhibited the growth of xenograft tumors (Fig. 6a), as evident by significantly reduced tumor weight in b-AP15-treated group compared to the vehicle-treated group (Fig. 6b). There was no significant difference in body weight (Fig. 6c), and the value of blood test indicators of hepatorenal function remained stable (Fig. 6d). The protein level of cleaved PARP, a hallmark of apoptosis, was markedly increased in b-AP-15-treated tumors (Fig. 6e). The ubiquitin-proteins was highly accumulated in tissue samples of b-AP15-treated tumors compared with the control group (Fig. 6f). Furthermore, protein biomarkers associated with proliferation and migration, such as c-Myc, Bcl-2, p65, Ki67, β-catenin and Smad2/3, were downregulated in b-AP15-treated tumors (Fig. 6e and f). These results demonstrated that b-AP15 inhibited the growth of GCB- and ABC-DLBCL xenografts.

## Discussion

The ubiquitin proteasome pathway has been validated as a novel therapeutic target in cancer. The first proteasome inhibitor, bortezomib, has been approved by the US FDA as a single agent or in combination in multiple myeloma. Recent preclinical and clinical studies demonstrated that targeting the canonical NF-κB pathway through inhibition of the 20S proteasome with bortezomib could kill DLBCL cells [32, 33]. Unfortunately, not all DLBCL are bortezomib-sensitive, and patients may eventually develop bortezomib-resistant disease [34]. It has been reported that USP14 and UCHL5 are involved in the development of tumor and are potential new targets for proteasome inhibition in DLBCL [22]. In the current study we planned to figure out whether b-AP15 could inhibit the progression of DLBCL, and we report that b-AP15 can do so through inhibiting the deubiquitinases activities of USP14 and UCHL5.

We found that b-AP15, a novel molecule inhibitor of USP14 and UCHL5 [23], significantly inhibited the viability and induced apoptosis of GCB- and ABC-DLBCL cells. In addition, we also found that treatment with b-AP15 suppressed the migration of GCB- and ABC-DLBCL cells. Results from nude mouse xenograft models of two types DLBCLs also showed that b-AP15 inhibited tumor growth in vivo.

Our study revealed that b-AP15-induced apoptosis was associated with caspase activation and mitochondria apoptosis (Figs. 1 and 2). b-AP15 downregulates the protein level of XIAP, Bcl-1, Bcl-xl and Survivin. The altered ratio of anti-apoptosis and pro-apoptosis proteins triggered potential reduction in mitochondria, resulting in cytochrome C and AIF release and caspase activation and cell death.

We next investigated the mechanism underlying pro-apoptotic activity of b-AP15. We showed that b-AP15 induced a rapid and significant accumulation of ubiquitin-proteins and substrate protein p27 and b-AP15 has no marked influence on peptidases of 20S proteasome (Fig. 3). In a short period of time, b-AP15 inhibited the function of proteasome, following by the cleavage of PARP. Recent reports have identified that b-AP15 treatment led to the accumulation of misfolded proteins to trigger ER stress [35]. It is a widely accepted concept that ER stress can activate caspase pathway and induce cell apoptosis [36]. We speculated that b-AP15 targeted the DUB function of USP14 and UCHL5, a large amount of unfolded proteins triggered ER stress to induce the cell apoptosis. On the other hand, our study showed that b-AP15 distinctly downregulated those proteins associated with cancer progression in ABC- and GCB-DLBCLs (Figs. 4 and 5). We detected the mRNA and protein levels of p65, Bcl-2 and c-Myc, and the results showed that both the mRNA and protein levels were all decreased except the protein level of Bcl-2. Together, these data may explain the growth and migration inhibition as well as apoptosis induction effects of b-AP15 on both ABC- and GCB-DLBCL.

It is well established that metastasis is an important cause for highly lethality. Recent studies showed that USP14 is overexpressed in colorectal cancer and esophageal squamous cell carcinoma (ESCC) [18, 37]. Downregulation of USP14 resulted in accumulation of poly-ubiquitinated forms of Dvl, which significantly impairs downstream Wnt signaling [30]. The HA-Ub-VS assay demonstrates that b-AP15 inhibits the deubiquitinase activity of both USP14 and UCHL5. b-AP15 treatment induces the decreases of Dvl, β-catenin and c-Myc resulting in inhibition of Wnt signaling and the cell migration of ABC- and GCB-DLBCL cells (Fig. 4a and b). Our data shows the cell migration was activated by SKL2001 (Wnt/β-catenin signaling activator) and decreased by IWR-1-endo (β-catenin pathway inhibitor). Meanwhile, the inhibition of b-AP15 in cell migration was antagonized by SKL2001 (Fig. 4d and e), showing that the Wnt/β-catenin signaling plays an important role in regulating DLBCL cells migration. Like USP14, UCHL5 is also involved in tumorigenesis and progression [38]. It has been reported that UCHL5 combined with transcription factor Smad2/3, can regulate TGFβ signaling [38, 39]. Our result illustrates that b-AP15 decreases the protein level of Smad2/3, and the phosphorylated Smad2/3 (Fig. 4c). Furthermore, both SIS3 HCl and TP0427735 HCl (TGFβ/Smad signaling inhibitors) exhibit a suppression function on both p-Smad 2/3 protein level (Fig. 4f) and cell migration (Fig. 4g) in DLBCL cells, indicating that inhibiting TGFβ/Smad pathway could inhibit cell migration of DLBCL cells, which is similar to b-AP15 function. Moreover, we observe that TGFβ1 induces p-Smad 2/3 and partially rescues the inhibition of p-Smad 2/3 by b-AP15 (Fig. 4f), while its effect on the proportion of cell migration is not significant. Taken together, these findings suggest that b-AP15-regulated cell migration in DLBCL cells is associated with Wnt/β-catenin and TGFβ/Smad signaling pathways, whereas the Wnt/β-catenin signaling pathway may play a more important role in b-AP15 regulated cell migration. Besides, the suppressing function of b-AP15 in cell migration has been confirmed in vitro but should be further investigated in vivo.

## Conclusion

In conclusion, our research confirmed that b-AP15 inhibits the activity of two proteasomal DUBs, USP14 and UCHL5, leading to induce ABC- and GCB-DLBCL cell apoptosis. b-AP15 also inhibits Wnt and TGFβ signaling pathways and suppresses ABC- and GCB-DLBCL cells migration. Our studies on the basic research of b-AP15 suggest the feasibility of the clinical application of b-AP15 in DLBCLs.

## Supplementary information

**Additional file 1: Figure S1.** The cell apoptosis was detected with the treatment of lower concentration of b-AP15. Flow cytometry assay was shown after Annexin V-FITC / PI double staining.

## Data Availability

Detail reagents are available from the corresponding author upon reasonable request.

## Abbreviations

ABC: Activated B cell-like
CHOP: Cytoxan, hydroxyrubicin, oncovin, and prednisone
DLBCL: Diffuse large B cell lymphoma
DMSO: Dimethyl sulfoxide
DUBs: Deubiquitinases
GCB: Germinal center B cell-like
HRP: Horseradish peroxidase
IHC: Immunohistochemical staining
MM: Multiple myeloma
PARP: Poly adenosine diphosphate ribose polymerase
PMBCL: Primary mediastinal B cell lymphoma
PMSF: Phenylmethylsulfonyl fluoride
