# Supplementary figures and images for: Proteasomal cysteine deubiquitinase inhibitor b-AP15 suppresses migration and induces apoptosis in diffuse large B cell lymphoma
Source: J Exp Clin Cancer Res. 2019 Nov 6;38:453. doi: 10.1186/s13046-019-1446-y (PMC6836452; doi:10.1186/s13046-019-1446-y)

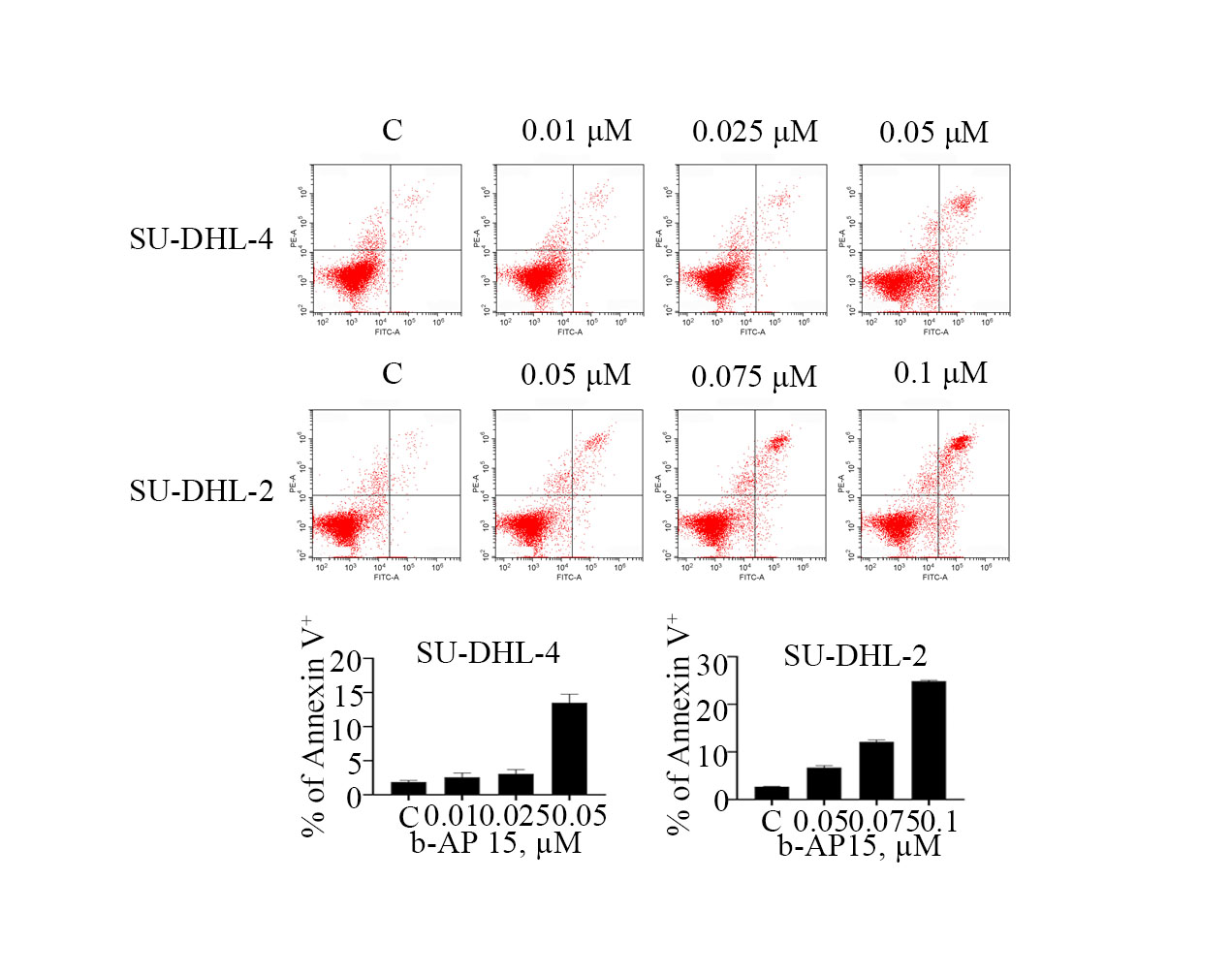

Supplement: Supplementary file 1 — Additional file 1: Figure S1. The cell apoptosis was detected with the treatment of lower concentration of b-AP15. Flow cytometry assay was shown after Annexin V-FITC / PI double staining. [file 13046_2019_1446_MOESM1_ESM.jpg]
